# 4164 Body mass index, not chemotherapy is the major predictor of insulin resistance in patients with hematological malignancies

**DOI:** 10.1017/cts.2020.372

**Published:** 2020-07-29

**Authors:** Melis Sahinoz, Brian Engelhardt, Dae Kwang Jung, James Matthew Luther, Talat Alp Ikizler

**Affiliations:** 1 Vanderbilt University Medical Center

## Abstract

OBJECTIVES/GOALS: Cancer survivors are at increased risk for type 2 diabetes mellitus.It is not clear if diabetes susceptibility is due to shared risk factors for cancer and diabetes, such as obesity, or if it is directly related to cancer and its treatment. We investigated the association between malignancy and insulin resistance, a major risk factor for diabetes. METHODS/STUDY POPULATION: 20 adult patients with treated hematological malignancies and 21 controls without cancer were included in the study. Individuals with pre-existing diabetes were excluded. All patients underwent a 2-hour 75-gram oral glucose tolerance test (OGTT). Hyperinsulinemic-euglycemic clamps were performed to measure the steady-state glucose infusion rate (M-value) as an indicator of whole-body glucose utilization during insulin stimulation. Insulin sensitivity index was calculated by dividing M-value over the steady-state plasma insulin (M/I). RESULTS/ANTICIPATED RESULTS: Fasting or postprandial plasma glucose levels during the OGTT did not differ significantly between malignancy patients and controls (Table 2). Difference in the insulin-stimulated glucose utilization (M-value) was not statistically significant among cancer patients and controls (median, 7.2 [IQR, 6.2-10.4] vs. 7.3 [IQR, 5.5-8.9] mg/kg/min; P = 0.261). M/I index was significantly higher in malignancy patients compared to controls (median, 42.4 mg/kg/min/(µU/ml) [IQR, 33.9-67.2] vs. 23.4 mg/kg/min/(µU/ml) [IQR, 12.9-29.2], P <0.001), however insulin clearance was also lower in the controls. In multivariate analysis, only BMI was significantly associated with M-value (β = −0.2 (95% CI −0.4, −0.1), P = 0.004), and M/I (β = −2 (95% CI −3.4, −0.5), P = 0.009). DISCUSSION/SIGNIFICANCE OF IMPACT: Our data suggest that the major contributor to diabetes development after diagnosis of cancer in adults is obesity-induced insulin resistance, not malignancy related factors. These findings emphasize the importance of obesity management in long-term survival of cancer patients, as diabetes is a risk factor for mortality in this patient population.

